# Replication Study Confirms Link between *TSPAN18* Mutation and Schizophrenia in Han Chinese

**DOI:** 10.1371/journal.pone.0058785

**Published:** 2013-03-07

**Authors:** Jianmin Yuan, Chunhui Jin, Hai-De Qin, Jidong Wang, Weiwei Sha, Mingzhong Wang, Yunbiao Zhang, Fuquan Zhang, Jijiang Li, Jianfeng Li, Shui Yu, Shuguang Qi, Yin Yao Shugart

**Affiliations:** 1 Wuxi Mental Health Center, Wuxi, Jiangsu Province, China; 2 Jiangsu Institute of Psychology, Wuxi, Jiangsu Province, China; 3 Intramural Research Program, National Institute of Mental Health, National Institutes of Health, Bethesda, Maryland, United States of America; 4 Yangzhou Wutaishan Hospital, Yangzhou, Jiangsu Province, China; 5 Nanjing Qinglongshan Mental Hospital, Nanjing, Jiangsu Province, China; University of Hong Kong, Hong Kong

## Abstract

Schizophrenia (SCZ) is a severe psychiatric disorder associated with many different risk factors, both genetic and environmental. A recent genome-wide association study (GWAS) of Han Chinese identified three single-nucleotide polymorphisms (SNPs rs11038167, rs11038172, and rs835784) in the tetraspanins gene *TSPAN18* as possible susceptibility loci for schizophrenia. Hoping to validate these findings, we conducted a case-control study of Han Chinese with 1093 schizophrenia cases and 1022 healthy controls. Using the LDR-PCR method to genotype polymorphisms in *TSPAN18*, we found no significant differences (P>0.05) between patients and controls in either the allele or genotype frequency of the SNPs rs11038167 and rs11038172. We did find, however, that the frequency of the ‘A’ allele of SNP rs835784 is significantly higher in patients than in controls. We further observed a significant association (OR  = 1.197, 95%CI  = 1.047–1.369) between risk for SCZ and this ‘A’ allele. These results confirm the significant association, in Han Chinese populations, of increased SCZ risk and the variant of the *TSPAN18* gene containing the ‘A’ allele of SNP rs835784.

## Introduction

Schizophrenia (SCZ) – a severe and heritable psychiatric disorder characterized by positive and negative symptoms, some of which are cognitive – affects ∼1% of the general population worldwide. While the heritability of schizophrenia has been estimated to be ∼64–80% [1], [2], its precise etiology and genetic mechanisms remain unclear. One genome-wide association study (GWAS) associates susceptibility for SCZ with several common single-nucleotide polymorphisms (SNPs) and rare copy-number variants (CNVs). To date, however, these suspected susceptibility loci have been studied primarily in populations of European and American descent [3], [4], [5], [6], [7].

Interestingly, a recent GWAS study of Han Chinese associated increased susceptibility for SCZ with three SNPs (rs11038167, rs11038172 and rs835784) within the tetraspanins gene *TSPAN18* [8]. *TSPAN18* at 11p11.2 encodes one member of a large family of membrane proteins found in all multicellular eukaryotes with four transmembrane (tetraspanin) domains. Expressed widely and in diverse cell types, the tetraspanins appear to affect cellular penetration, adhesion, motility, and signal conduction [9], [10], [11]. Although *TSPAN18*′s role in the pathogenesis of SCZ remains unclear, the tetraspanin family's association with both bipolar disorder and SCZ [8], [12] suggests it may well be involved in increasing susceptibility for SCZ. In an attempt to confirm such a role, this study aims to investigate the association of SCZ with three SNPs in the *TSPAN18* gene in an independent population of Han Chinese descent.

## Materials and Methods

### Study Population

Our study sample, which includes subjects of Han descent, includes 1093 patients (396 women and 697 men aged 47.9±10.9 years at recruitment) and 1022 unrelated healthy controls (450 women and 572 men aged 44.8±10.2 years at recruitment) (Table 1).

**Table 1:** ****Demographic characteristics of study subjects.

Group	Case *n* (%)	Control *n* (%)
*Sex*		
Total	1093	1022
Female	396 (0.36)	450 (0.41)
Male	697 (0.64)	572 (0.52)
*Age*		
Range	16–75	18–77
Mean	47.9±10.9	44.8±10.2
10–19	5 (0.00)	2 (0.00)
20–29	71 (0.06)	93 (0.09)
30–39	139 (0.13)	175 (0.16)
40–49	371 (0.34)	420 (0.38)
50–59	352 (0.32)	256 (0.23)
60–69	145 (0.13)	70 (0.06)
70–79	10 (0.01)	6 (0.01)

In the patient sample, the diagnosis of schizophrenia was confirmed by two or more experienced psychiatrists using the *Structured Clinical Interview for DSM-IV* (SCID-I) and criteria set forth in the *Diagnostic and Statistical Manual of Mental Disorders*, Fourth edition (DSM-IV). Exclusion criteria included the presence of other mood or neurodevelopmental disorders, epilepsy, or mental retardation. For the selection of controls, professional psychiatrists, using the *Structured Clinical Interview for DSM-IV, Non-patients edition* (SCID-NP), interviewed members of an unrelated general population. Subjects with mental illness within the SCID-I Axis I were excluded.

This study was approved by the Ethics Committees of the Wuxi Health Mental Center. Either patients or their guardians signed informed consents. Where ability to consent appeared compromised, we used the following criteria to evaluate whether the participants had the capacity to consent: 1) patient's ability to understand; 2) patient's ability to reason; and 3) patient's ability to make rational decisions. If participants failed to fill out the consent form more than twice, their guardians were asked to fill out the consent form on the patients' behalf.

Healthy subjects were recruited through advertisement. We recruited controls from the cities of WuXi and NanJing in Jiangsu Province. Based on self-report regarding their own and their paternal grandparents' place of birth, we excluded anyone not born in Jiangsu or whose family was not born in Jiangsu. Before being enrolled in the study, each healthy subject was required to sign a consent form.

### DNA extraction

Blood samples were collected from all participants using K_2_EDTA tubes. A Blood Genotyping DNA Extraction Kit (Tiangen Biotech, Beijing, China) was used to extract genomic DNA from 150 μl of peripheral blood. DNA samples were then stored at −80°C for genotype analysis.

### SNP genotyping

The genotype of each SNP was analyzed by the Shanghai Biowing Applied Biotechnology Co., Ltd (www.biowing.com.cn) using the Ligase Detection Reaction-Polymerase Chain Reaction (LDR-PCR) method [13], [14]. Genomic DNA extracted from clinical samples was first subjected to multiplex RCR to obtain a PCR product, including SNPs. This PCR product and LDR probes were then subjected to multiplex LDR reaction with a DNA sequencer to detect the products. To test the validity of this procedure, approximately 10% of the samples was randomly selected and retested using the same process. Results from the retested 10% were consistent with those obtained from the larger sample.

### Statistical analysis

Our statistical analyses, performed using PLINK software (http://pngu.mgh.harvard.edu/~purcell/plink), included association studies, Hardy-Weinberg equilibrium (HWE) tests, and the calculation of genotype and allele frequencies in schizophrenia patients and healthy controls. We also used a logistic regression model adjusted for age and sex to evaluate how these factors influence the distribution of *TSPAN18* polymorphisms. Frequency comparisons among different ethnic groups were conducted based on variation data from dbSNP build 135, which includes data from the HapMap and 1000-Genome projects.

## Results

This study analyzes data from 1093 patients diagnosed with schizophrenia and 1022 unrelated healthy controls. Our HWE tests indicate that the allelic frequency distribution of *TSPAN18* polymorphisms does not deviate significantly from the Hardy-Weinberg equilibrium (*P* = 0.1688 for rs11038167, 0.3681 for rs11038172, and 0.2972 for rs835784). The total genotyping rate in all individuals was 99.57%.

Results from our association study indicate a significant association (trend test: *P* = 0.004967; χ^2^ test under dominant model: *P* = 0.008812) between SCZ and the minor allele (‘A’) of the *TSPAN18* gene SNP rs835784. Stratified analysis by sex and age supports this association, with the χ^2^ test showing *P* = 0.01374 in males and *P* = 0.00339 in subjects of middle age (30–59 years). In this Chinese Han population, therefore, the minor allele ‘A’ of rs835784 appears to be a risk factor for SCZ (see Tables 2 and 3).

**Table 2:** **** Association study of three SNPs in *TSPAN18* under different models.

	Case		Control		
Test Model: SNP(A1/A2)†	*n*	Freq.§	*n*	Freq.§	*P* *
*rs11038167 (A; C)*					
Trend: A/C	877/1307	0.40	806/1234	0.40	0.6774
Allelic: A/C	877/1307	0.40	806/1234	0.40	0.6683
Dominant: (AA+AC)/CC	681/411	0.62	636/384	0.62	0.9963
Recessive: AA/(AC+CC)	196/896	0.18	170/850	0.17	0.4367
*rs11038172 (A; G)*					
Trend: A/G	965/1207	0.44	862/1158	0.43	0.2587
Allelic: A/G	965/1207	0.44	862/1158	0.43	0.252
Dominant: (AA+AG)/GG	743/343	0.68	671/339	0.66	0.3335
Recessive: AA/(AG+GG)	222/864	0.20	191/819	0.19	0.3786
*rs835784 (A;G)*					
Trend: A/G	667/1513	0.31	543/1497	0.27	0.004967
Allelic: A/G	667/1513	0.31	543/1497	0.27	0.00429
Dominant: (AA+AG)/GG	558/532	0.51	464/556	0.45	0.008812
Recessive: AA/(AG+GG)	109/981	0.10	79/941	0.08	0.06924

† A1/A2, indicates minor allele/major allele.

§ The minor allele frequency for allelic and trend model, “DD + Dd” frequency for dominant model, and “DD” for recessive model, where “D” indicates minor allele, “d” indicates the major allele.

* Cochran-Armitage trend test p-value; for Allelic/Dominant/Recessive models, asymptotic p-values were calculated by Chi-Squared test.

**Table 3:** llele frequency distribution by sex and age group for three SNPs in *TSPAN18.*

			Case		Control		
Group	SNP	A1/A2†	n	Freq. §	n	Freq. §	P*
Male							
	rs11038167	A/C	554/838	0.40	429/713	0.38	0.2725
	rs11038172	A/G	613/769	0.44	467/673	0.41	0.09324
	rs835784	A/G	420/968	0.30	293/849	0.26	0.01374
Female							
	rs11038167	A/C	323/469	0.41	377/521	0.42	0.6192
	rs11038172	A/G	352/438	0.45	395/485	0.45	0.893
	rs835784	A/G	247/545	0.31	250/648	0.28	0.1251
Younger (−29)							
	rs11038167	A/C	53/99	0.35	91/99	0.48	0.02013
	rs11038172	A/G	58/94	0.38	97/93	0.51	0.02125
	rs835784	A/G	39/113	0.26	61/129	0.32	0.2074
Middle-aged (30–59)							
	rs11038167	A/C	678/1044	0.39	657/1041	0.39	0.6924
	rs11038172	A/G	756/960	0.44	703/977	0.42	0.1983
	rs835784	A/G	523/1201	0.30	438/1262	0.26	0.00339
Elderly (60-)							
	rs11038167	A/C	146/164	0.47	58/94	0.38	0.07142
	rs11038172	A/G	151/153	0.50	62/88	0.41	0.09996
	rs835784	A/G	105/199	0.35	44/106	0.29	0.2691

† A1/A2, indicates minor allele/major allele.

§ The minor allele frequency.

* Cochran-Armitage trend test p-values for minor allele.

Using logistic regression models to estimate the effect size of the risk allele, we calculated that, before adjustment for age and sex, the OR for increased risk for SCZ with the rs835784 ‘A’ allele is 1.197 (95%CI = 1.047–1.369), *P* = 0.008426. Once adjusted for age and sex, the risk is nearly the same, OR  = 1.194 (95%CI = 1.033–1.381, *P* = 0.0165), confirming that the ‘A’ allele of rs835784 is consistently associated with an increase in risk for SCZ (Table 4).

**Table 4:** Risk estimates using logistic regression model for three SNPs in *TSPAN18.*

		All (*n* = 2,115)		All (*n* = 2,115)		Matched† (*n* = 1,804)	
SNP	Risk Allele	OR (95%CI)	*P_unadj_* ζ	OR (95%CI)	*P*_adj_ *	OR (95%CI)	*P*_adj_ *
rs11038167	*A*	1.026 (0.91–1.156)	0.6774	1.032 (0.9137–1.165)	0.6147	1.04 (0.9118–1.186)	0.5595
rs11038172	*A*	1.072 (0.9502–1.209)	0.2588	1.071 (0.948–1.21)	0.2696	1.083 (0.9487–1.236)	0.2384
rs835784	*A*	1.208 (1.059–1.379)	0.005034	1.197 (1.047–1.369)	0.008426	1.194 (1.033–1.381)	0.0165

† Sex- and age- (±2 year-old) matched dataset.

ζ *P*_unadj_, un-adjusted p-values in the logistic regression model.

* *P*_adj_, p-values of the risk allele in the logistic regression model, adjusted by sex and age.

By contrast, neither genotypic nor allelic modeling revealed significant association between increased schizophrenia risk and the other two *TSPAN18* SNPs, rs11038167 or rs11038172 (Table 2). Although stratified analysis does suggest that the ‘A’ alleles of both rs11038167 (*P* = 0.02013 in the χ^2^ test) and rs11038172 (*P* = 0.02125) may be associated with increased SCZ risk in youth (≤29-year-old) (Table 3), our sample (N = 190) was too small for these results to be reliable, and results adjusted for age and sex fail to support them (Table 4). In addition, we found an inter-marker LD relationship between these two SNPs and rs835784, whose A-allele appears to be associated with SCZ, with r-square = 0.45 for rs835784′s relationship with rs11038167 and r-square = 0.38 for its relationship with rs11038172.

## Discussion and Conclusions

The tetraspanins are a highly conserved superfamily of cell-surface membrane proteins known to be influential in diverse diseases and physiologic processes. High expression of TSPAN1, TSPAN8, or TSPAN2, for example, which are all in the same protein family as *TSPAN18*, correlates positively with tumor progression [15], [16], [17], while mutation in TSPAN7 is associated with X-linked mental retardation [18], [19].

Although earlier reports implicate *TSPAN18* in susceptibility for SCZ [8], Ma et al. [20] failed to confirm that association. In this case-control study of Han Chinese, we, too, find no statistically significant association between SCZ and the two *TSPAN18* SNPs rs11038167 and rs11038172. Our analyses do reveal a statistically significant difference between patients with schizophrenia and healthy controls: the frequency of the “A” allele of rs835784 occurs at 31% in our SCZ samples and at 27% in controls. This finding confirms Yue et al.'s [8] report that carriers of the A-allele of rs835784 are at 1.197-fold greater risk for SCZ than non-carriers.

It should be noted that this study's subjects were all recruited from Jiangsu province, while Yue et al.'s study sample was drawn from northern China (an area that includes Beijing, Tianjin, Hebei and Shandong), and Ma et al.'s from Hunan province. It is therefore possible that differences in their *TSPAN18* polymorphism profiles could reflect regional differences not generalizable to all people of Han descent.

**Figure 1:**
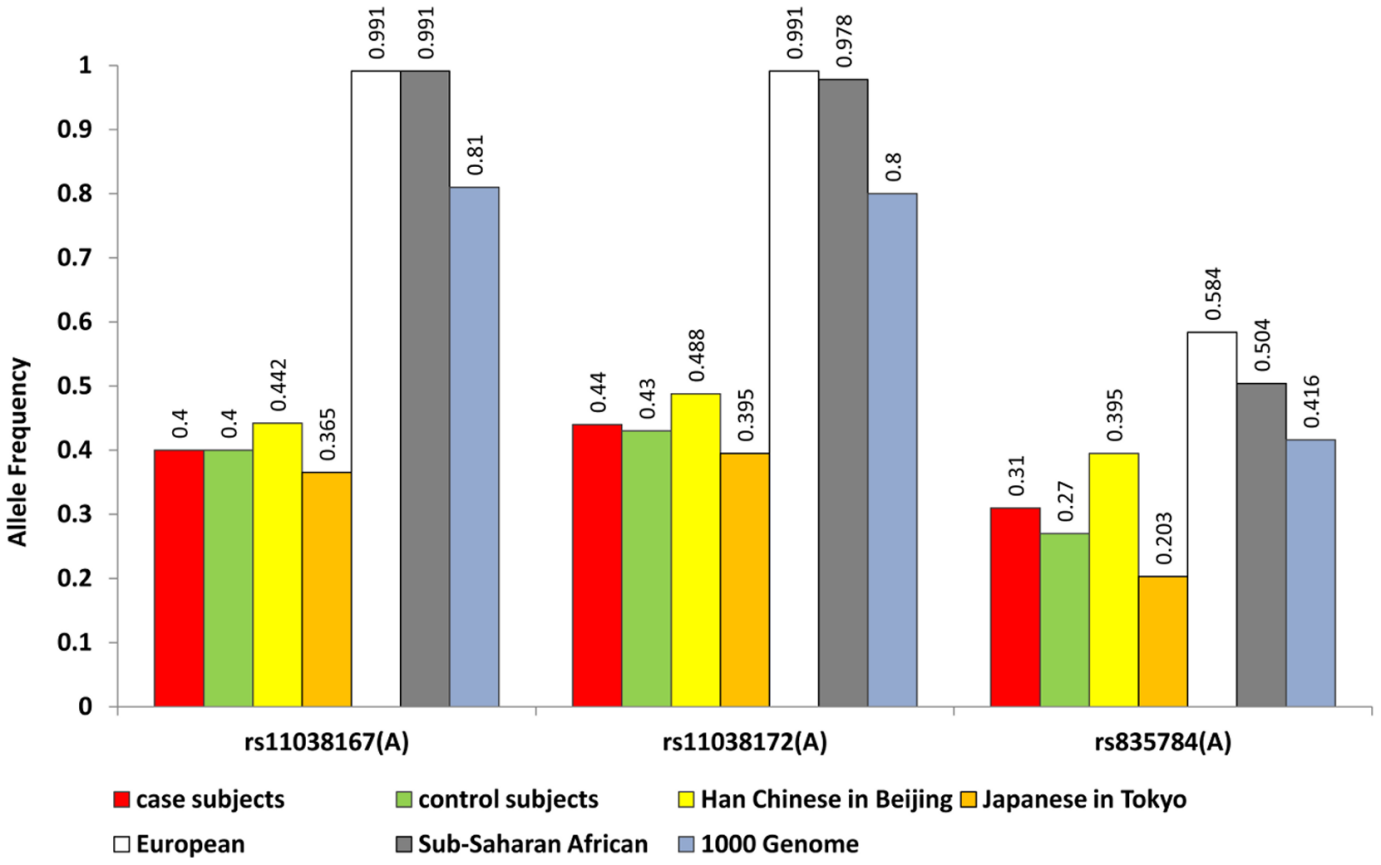
Allele frequency distribution among different ethnic groups for the three SNPs in *TSPAN18.*

While a more definitive assessment of potential population stratification among Han Chinese subpopulations is not feasible in this study, we were able to compare SNP frequency distribution in different ethnic groups using dbSNP data from HapMap and the 1000-genomes studies. Our allele frequency analysis of these data for the three *TSPAN18* SNPs revealed substantial differences among Asian, European, and African populations (Figure 1). To identify precisely how ethnicity and geographic origin affect the distribution of genetic factors associated with schizophrenia, larger replication studies are needed.

To summarize, this case-control study of a Han Chinese population validated a SNP locus in *TSPAN18* reported by an earlier GWAS study and provided important evidence for the association of a particular variant of the *TSPAN18* gene with increased SCZ susceptibility. Further studies to validate these findings on the molecular level are clearly warranted. Data from this study are available upon request.

## References

[1] Lichtenstein P, Yip BH, Bjork C, Pawitan Y, Cannon TD, et al (2009) Common genetic determinants of schizophrenia and bipolar disorder in Swedish families: a population-based study. Lancet 373: 234–239. 19150704 10.1016/S0140-6736(09)60072-6 PMC3879718

[2] Thaker GK, Carpenter WT (2001) Advances in schizophrenia. Nat Med 7: 667–671. 11385502 10.1038/89040

[3] O'Donovan MC, Craddock N, Norton N, Williams H, Peirce T, et al (2008) Identification of loci associated with schizophrenia by genome-wide association and follow-up. Nat Genet 40: 1053–1055. 18677311 10.1038/ng.201

[4] Stefansson H, Ophoff RA, Steinberg S, Andreassen OA, Cichon S, et al (2009) Common variants conferring risk of schizophrenia. Nature 460: 744–747. 19571808 10.1038/nature08186 PMC3077530

[5] Shi J, Levinson DF, Duan J, Sanders AR, Zheng Y, et al (2009) Common variants on chromosome 6p22.1 are associated with schizophrenia. Nature 460: 753–757. 19571809 10.1038/nature08192 PMC2775422

[6] Purcell SM, Wray NR, Stone JL, Visscher PM, O'Donovan MC, et al (2009) Common polygenic variation contributes to risk of schizophrenia and bipolar disorder. Nature 460: 748–752. 19571811 10.1038/nature08185 PMC3912837

[7] Vacic V, McCarthy S, Malhotra D, Murray F, Chou HH, et al (2011) Duplications of the neuropeptide receptor gene VIPR2 confer significant risk for schizophrenia. Nature 471: 499–503. 21346763 10.1038/nature09884 PMC3351382

[8] Yue WH, Wang HF, Sun LD, Tang FL, Liu ZH, et al (2011) Genome-wide association study identifies a susceptibility locus for schizophrenia in Han Chinese at 11p11.2. Nat Genet 43: 1228–1231. 22037552 10.1038/ng.979

[9] Hemler ME (2003) Tetraspanin proteins mediate cellular penetration, invasion, and fusion events and define a novel type of membrane microdomain. Annu Rev Cell Dev Biol 19: 397–422. 14570575 10.1146/annurev.cellbio.19.111301.153609

[10] Tarrant JM, Robb L, van Spriel AB, Wright MD (2003) Tetraspanins: molecular organisers of the leukocyte surface. Trends Immunol 24: 610–617. 14596886 10.1016/j.it.2003.09.011

[11] Levy S, Shoham T (2005) The tetraspanin web modulates immune-signalling complexes. Nat Rev Immunol 5: 136–148. 15688041 10.1038/nri1548

[12] Scholz CJ, Jacob CP, Buttenschon HN, Kittel-Schneider S, Boreatti-Hummer A, et al (2010) Functional variants of TSPAN8 are associated with bipolar disorder and schizophrenia. Am J Med Genet B Neuropsychiatr Genet 153B: 967–972. 20052686 10.1002/ajmg.b.31057

[13] O’Donovan MC, Craddock N, Norton N, Williams H, Peirce T, et al (2008) Identification of loci associated with schizophrenia by genome-wide association and follow up. Nat Genet 40: 1053–1055. 18677311 10.1038/ng.201

[14] Shi Y, Li Z, Xu Q, Wang T, Li T, et al (2011) Common variants on 8p12 and 1q24.2 confer risk of schizophrenia. Nat Genet 43: 1224–1227. 22037555 10.1038/ng.980 PMC3773910

[15] Zhou Z, Ran YL, Hu H, Pan J, Li ZF, et al (2008) TM4SF3 promotes esophageal carcinoma metastasis via upregulating ADAM12m expression. Clin Exp Metastasis 25: 537–548. 18365756 10.1007/s10585-008-9168-0

[16] Yang XH, Richardson AL, Torres-Arzayus MI, Zhou P, Sharma C, et al (2008) CD151 accelerates breast cancer by regulating alpha 6 integrin function, signaling, and molecular organization. Cancer Res 68: 3204–3213. 18451146 10.1158/0008-5472.CAN-07-2949 PMC4764302

[17] Scholz CJ, Kurzeder C, Koretz K, Windisch J, Kreienberg R, et al (2009) Tspan-1 is a tetraspanin preferentially expressed by mucinous and endometrioid subtypes of human ovarian carcinomas. Cancer Lett 275: 198–203. 19017553 10.1016/j.canlet.2008.10.014

[18] Zemni R, Bienvenu T, Vinet MC, Sefiani A, Carrie A, et al (2000) A new gene involved in X-linked mental retardation identified by analysis of an X;2 balanced translocation. Nat Genet 24: 167–170. 10655063 10.1038/72829

[19] Maranduba CM, Sa Moreira E, Muller Orabona G, Pavanello RC, Vianna-Morgante AM, et al (2004) Does the P172H mutation at the TM4SF2 gene cause X-linked mental retardation? Am J Med Genet A 124A: 413–415. 14735593 10.1002/ajmg.a.20401

[20] Ma L, Tang J, Wang D, Zhang W, Liu W, et al. (2012) Evaluating risk loci for schizophrenia distilled from genome-wide association studies in Han Chinese from central China. Mol Psychiatry 1–2. 10.1038/mp.2012.63 22584866

